# Genetic structure of colline and montane populations of an endangered plant species

**DOI:** 10.1093/aobpla/plw057

**Published:** 2016-10-19

**Authors:** Tiphaine Maurice, Diethart Matthies, Serge Muller, Guy Colling

**Affiliations:** 1 Université de Lorraine, CNRS UMR 7360, Laboratoire Interdisciplinaire des Environnements Continentaux (LIEC), rue du Général Delestraint, F-57070 Metz, France; 2 Musée National d'Histoire Naturelle, Population Biology and Evolution, 25 rue Münster, L-2160 Luxembourg, Luxembourg; 3 Fondation Faune Flore, 24 rue Münster, L-2160 Luxembourg, Luxembourg; 4 Philipps-Universität, Fachbereich Biologie, Pflanzenökologie, D-35032 Marburg, Germany; 5 Muséum national d'Histoire naturelle, UMR 7205 ISYEB, CNRS, Université Pierre-et-Marie-Curie, EPHE, Sorbonne Universités, CP 39, 16 rue Buffon, F-75005 Paris, France

**Keywords:** AFLP, altitude, clonality, conservation genetics, fragmentation, genome scan

## Abstract

Due to land-use intensification, lowland and colline populations of many plants of nutrient-poor grasslands have been strongly fragmented in the last decades, with potentially negative consequences for their genetic diversity and persistence. Populations in mountains might represent a genetic reservoir for grassland plants, because they have been less affected by land-use changes. We studied the genetic structure and diversity of colline and montane Vosges populations of the threatened perennial plant *Arnica montana* in western central Europe using AFLP markers. Our results indicate that in contrast to our expectation even strongly fragmented colline populations of *A. montana* have conserved a considerable amount of genetic diversity. However, mean seed mass increased with the proportion of polymorphic loci, suggesting inbreeding effects in low diversity populations. At a similar small geographical scale, there was a clear IBD pattern for the montane Vosges but not for the colline populations. However, there was a strong IBD-pattern for the colline populations at a large geographical scale suggesting that this pattern is a legacy of historical gene flow, as most of the colline populations are today strongly isolated from each other. Genetic differentiation between colline and montane Vosges populations was strong. Moreover, results of a genome scan study indicated differences in loci under selection, suggesting that plants from montane Vosges populations might be maladapted to conditions at colline sites. Our results suggest caution in using material from montane populations of rare plants for the reinforcement of small genetically depauperate lowland populations.

## Abstract (precis)

We found that populations of the threatened plant Arnica montana have conserved a considerable amount of genetic diversity, even when they have become fragmented. However, populations in the Vosges mountains differed strongly in their genetic makeup from populations at lower elevations. It has been suggested to reinforce lowland populations of rare plants with seeds from the uplands, but in view of our results this does not seem to be a good idea for A. montana. Because they are genetically different, plants from the mountains could be less able to cope with conditions in the lowlands than lowland plants.

## Introduction

Nutrient-poor grasslands in lowland areas have been strongly fragmented during the last decades due to changes in land-use, nutrient enrichment through fertilizers or the cessation of traditional agricultural practices (Ratcliffe 1984; Bignal and McCracken 1996). As a consequence, many formerly common grassland species have been reduced to small and isolated populations (see Fischer and Matthies 1998; Kéry *et al.* 2000; Colling and Matthies 2006). These populations face an increased risk of extinction because of their higher sensitivity to environmental, demographic and genetic stochasticity (Young *et al.* 1996; Matthies *et al*. 2004). Small and isolated populations are threatened through a loss of genetic diversity due to genetic drift and reduced gene flow (Young *et al.* 1996; Jacquemyn *et al*. 2009), because the loss of genetic variation and increased inbreeding are expected to lead to lower fitness of individual plants and a reduced ability of the populations to respond to environmental changes (Kéry and Matthies 2004; Ouborg *et al.* 2006; Walisch *et al.* 2012).

To enhance the chances of survival of small and isolated populations, it has been suggested to artificially augment threatened populations by introducing seeds from extant large populations to increase their size and genetic diversity (Ingvarsson 2001; Hufford and Mazer 2003; Tallmon *et al* 2004). European mountains could represent a genetic reservoir for plants of nutrient-poor grasslands as the intensification of land-use affecting lowland areas since several decades has only recently begun in mountain areas (Fischer and Wipf 2002; Peter *et al.* 2009). However, environmental conditions at higher altitudes such as low temperatures, a short growing season, strong winds, high irradiance, low air pressure and variation in the persistence of snow cover (Körner 2007) impose strong environmental constraints that can lead to marked genetic differences among plant populations along altitudinal gradients (Parker *et al*. 2003; Montesinos-Navarro *et al*. 2011). If populations at higher altitudes were locally adapted, this could present a problem for management measures such as the reinforcement of lowland populations with seeds from mountain populations, because the transplants could be maladapted (Vergeer *et al.* 2004; McKay *et al.* 2005). Moreover, crossings between strongly differentiated genotypes could result in outbreeding depression in the offspring (Hufford and Mazer 2003; Galloway and Etterson 2005; Walisch *et al*. 2012).

We studied the population genetic structure of the endangered long-lived grassland species *Arnica montana*, a characteristic species of acid nutrient-poor grasslands in Central Europe, in the colline Ardennes-Eifel and Hunsrück regions and the nearest montane region, the Vosges mountains. *A. montana* has strongly declined in lowland and colline regions and is now considered to be endangered in many parts of Europe (Korneck *et al.* 1996; Colling 2005; Kestemont 2010). Its decline has been attributed to the deterioration of habitat quality due to changes in land use, increased use of fertilizer and aerial deposition of nitrogen (Fennema 1992; Vergeer *et al.* 2005; Maurice *et al.* 2012). The remaining colline and lowland populations, but also populations in some mountain ranges are fragmented (Kahmen and Poschlod 2000; Luijten *et al*. 2000), while in other mountain ranges like the Vosges *A. montana* is still rather common and even harvested (Ellenberger 1998; Schnitzler and Muller 1998).

We asked the following questions: (1) Do the genetic diversity and genetic structure of the colline Ardennes-Eifel and Hunsrück populations and montane Vosges populations of *A. montana* differ, and in particular, (2) Are the colline populations genetically depauperate? (3) Are there differences between colline and Vosges populations in AFLP loci?

## Methods

### Study species

*Arnica montana* (*Asteraceae*) is a long-lived perennial plant that produces large rosettes from a rhizome. The species is restricted to Europe (Hultén and Fries 1996). *A. montana* can form dense mats that may consist of several different genotypes, and without genetic analyses it is not possible to distinguish individual genets (Luijten *et al.* 1996). *A. montana* has a sporophytic self-incompatibility system (Luijten *et al.* 2002). The large orange-yellow flowerheads of *A. montana* produce achenes (hereafter called seeds), which are wind-dispersed. Although the seeds of *A. montana* are small (mass c. 1.3 mg) and possess a pappus, their dispersal is very limited (Luijten *et al.* 1996; Strykstra *et al.* 1998). *A. montana* is an important source of pharmaceutical compounds (Lyss *et al*. 1997; Klaas *et al*. 2002) and the species is still harvested in some mountain regions due to difficulties in cultivating it (Delabays and Mange 1991; Mardari *et al*. 2015).

### Study area and sampling procedure

**Figure 1.**
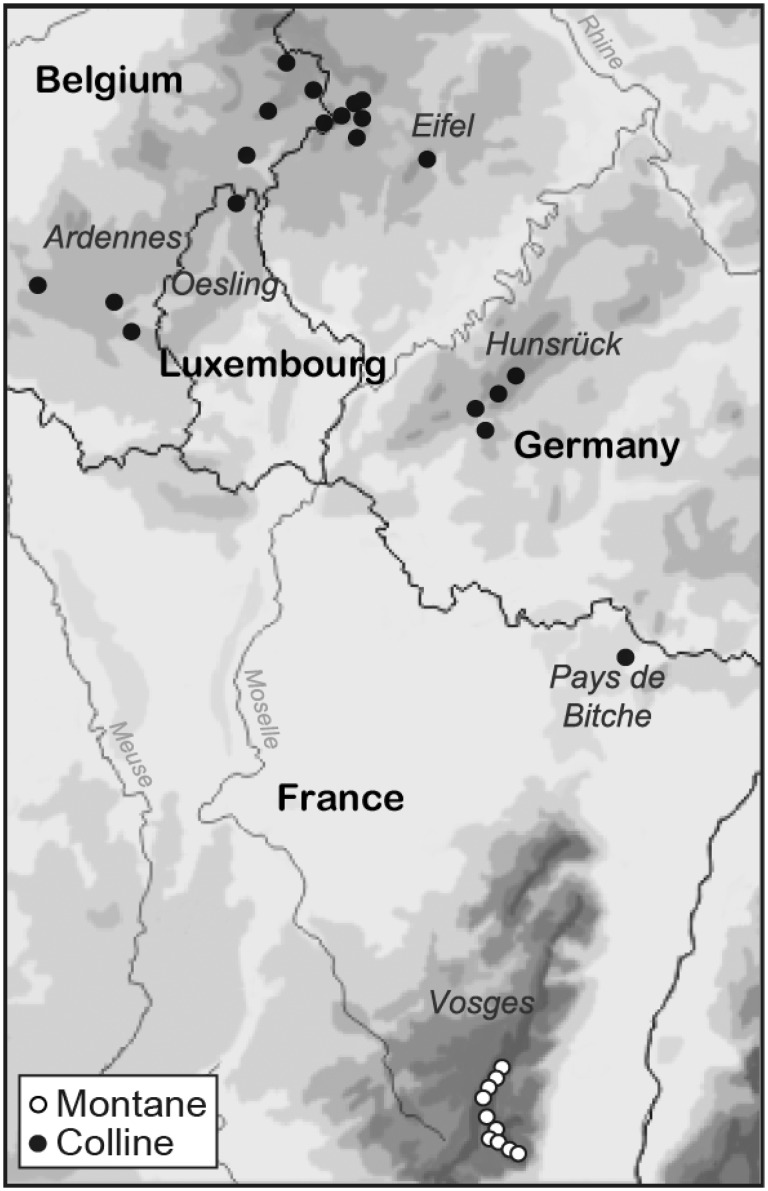
Map showing the location of the studied montane populations of *A. montana* in the Vosges mountains (open circles) and of the colline populations (filled circles) in the Ardennes–Eifel, Hunsrück and Pays de Bitche regions.

To study the genetic structure of *A. montana* in Western-Central Europe, samples were taken in 30 populations of different sizes in three neighbouring geographical regions: (1) The colline region of the Ardennes (Belgium), the Oesling (Luxembourg) and the Eifel (Germany), (2) the colline region of the Hunsrück (Germany) with the neighbouring Pays de Bitche region (France) and (3) the montane belt of the French Vosges mountains, which is the mountain range nearest to the colline study area (Fig. 1 and Table 1). Apart from altitude, the colline and montane populations differ in the composition of the vegetation and, based on Ellenberg indicator values calculated from the vegetation data, in soil moisture, but not in soil reaction and soil nutrients (Maurice *et al.* 2012).

**Table 1.** Characteristics of the 30 studied populations of *A.* *montana*. Region, geographical region (see text for details); pop. Name, population name; pop. size, population size calculated as total number of rosettes (see methods for details); *n*, number of rosettes analysed genetically. In some populations (in italics) a lower number of rosettes was analysed due to PCR problems (see text for details). P, proportion of polymorphic loci; H_e_, Nei's expected heterozygosity based on allele frequencies calculated by the square root method, assuming Hardy–Weinberg equilibrium; Cl, STRUCTURE cluster ID: AE, Ardennes–Eifel; H, Hunsrück; V, Vosges mountains. The proportion of individuals assigned to the clusters is indicated as subscript.

Region	Pop. name	Alt. (m)	Pop. size	Latitude (^o^ North)	Longitude (^o^ East)	*n*	P (%)	H_e_	Cl
Ardennes	A-Bas	449	11	49.833	5.606	11	60.9	0.236	AE_1.00_
− Eifel	A-Jus	448	340	49.888	5.549	20	72.4	0.236	AE_1.00_
	A-Roc	418	670	49.920	5.238	20	72.4	0.239	AE_1.00_
	*A-Tho*	496	270	50.239	6.001	9	76.2	0.229	AE_1.00_
	*A-Em*	609	150	50.326	6.405	6	65.9	0.222	AE_1.00_
	A-Els	591	18000	50.454	6.259	20	84.0	0.240	AE_1.00_
	A-Sch	457	1100	50.225	6.049	20	86.7	0.248	AE_1.00_
	A-Kap	485	110	50.311	6.142	11	73.4	0.252	AE_1.00_
	O-Lux	516	260	50.146	6.036	15	74.4	0.247	AE_1.00_
	E-Auf	537	710	50.364	6.534	20	83.5	0.236	AE_0.95_
	E-Asb	538	10000	50.364	6.524	20	78.2	0.205	AE_1.00_
	E-Leu	598	4000	50.349	6.484	20	74.4	0.211	AE_0.95_
	E-Ste	584	7600	50.329	6.558	20	74.2	0.206	AE_1.00_
	E-Man	538	2100	50.291	6.540	20	75.9	0.221	AE_1.00_
	E-Dau	481	2300	50.240	6.826	20	81.7	0.232	AE_1.00_
Hunsrück	H-Abe	496	570	49.657	7.094	20	61.4	0.174	H_1.00_
	H-Bra	558	1200	49.574	7.003	13	46.6	0.164	H_1.00_
	H-Eis	448	4200	49.612	7.049	19	55.6	0.168	H_1.00_
	H-Otz	410	1300	49.601	6.979	20	62.2	0.173	H_1.00_
	P-Bit	281	300	49.066	7.528	20	72.2	0.210	AE_0.90_
Vosges	V-Fer	1225	600	48.052	7.020	18	68.9	0.198	V_1.00_
	*V-Cha*	1251	5000	48.049	7.012	7	72.9	0.206	V_1.00_
	V-Hon	1243	260	48.040	7.008	18	68.2	0.203	V_1.00_
	V-Sch	1225	1400	48.034	6.996	17	68.7	0.209	V_1.00_
	*V-Her*	1223	1500	47.986	6.980	5	54.1	0.201	V_1.00_
	V-Hah	1223	2200	47.942	7.022	14	60.9	0.194	V_1.00_
	V-Ste	1214	170	47.931	7.018	20	60.2	0.182	V_1.00_
	V-Mar	1175	2800	47.924	7.034	18	57.9	0.183	V_1.00_
	V-Moo	1218	4300	47.907	7.075	16	58.1	0.189	V_1.00_
	V-Haa	1268	450	47.904	7.093	17	59.1	0.187	V_1.00_

The studied 20 colline populations (281–633 m a.s.l.) represent a large part of the extant populations in the area. Although some large populations still occur in the colline region of the Ardennes-Eifel, many of the extant populations are small due to small habitat size and low habitat quality, in particular high nutrient levels which are known to be detrimental to *A. montana* (Vergeer *et al.* 2005, Maurice *et al.* 2012). The geographical distance between most extant populations in the colline area was large due to the intense habitat fragmentation, and ranged from 0.7 km to 190.7 km (median  =  77.9 km).

Ten montane populations were sampled in the French Vosges mountains (1175–1268 m a.s.l., Fig. 1). The populations were sampled in the part of the Vosges with the highest density of populations of *A. montana*. The geographical distance between montane populations was much smaller than that between colline populations (0.7–17.3 km; median  =  8.0 km).

Population sizes were estimated as the total number of rosettes (ramets) per population as it is not possible to distinguish individual genets in the field due to the clonal growth of *A. montana*. Based on the results of a former study of the structure of *A. montana* populations (Maurice *et al.* 2012), ramet population size was calculated by dividing the number of flowering stems by the proportion of flowering rosettes per population.

In June 2007, we collected one fresh leaf from each of 20 rosettes in each population along transects of 20 m length. Within each transect, we recorded the distances among the sampled plants. Because *A. montana* is a clonal species, the minimum distance between the sampled rosettes was 1 m to avoid sampling of the same genetic individual twice. In very small populations, the number of sampled rosettes was less than 20 (Table 1). The leaf samples were immediately stored in silica gel and kept at room temperature until DNA extraction. In several populations, we had problems with the PCR-reaction, probably due to the high content of secondary metabolites in the leaf tissue of *A. montana* (Ekenäs *et al.* 2009) and a lower number of samples were used for the genetic analyses (Table 1).

### DNA extraction, purification and AFLP analysis

A 96 wells DNeasy kit extraction (Qiagen®) was performed on 10 mg of dried leaf tissue for 494 samples after grinding (Retsch MM200, Retsch, Haan, Germany). Extracted DNA was purified from secondary metabolites with a ChargeSwitch® gDNA Plant Kit (Invitrogen®). A further purification step was done by electrophoresis on a 2 % agarose gel (90 V, 200 mA, 45 min) in 10X TBE buffer UltraPure (Invitrogen®, Tris 1 mM, Boric Acid 0.9 mM, EDTA 0.01 mM). Extraction of the samples from the gel was done using a QIAquick® 96 PCR Purification Kit (Qiagen®).

DNA (0.1 µg) was digested at 37 °C for 2 h using *Eco*RI and *Mse*I (1.3 U, Invitrogen®), 0.65 µL of 10X REact® 1 Buffer (Invitrogen®, 50 mM Tris-HCl, 10 mM MgCl_2_) and 0.65 µL of 10X REact® 3 Buffer (Invitrogen®, 50 mM Tris-HCl, 10 mM MgCl2, 100 mM NaCl) in a final volume of 10 µL. Endonucleases were inactivated by 15 min at 70 °C. Adaptor ligation was achieved by adding 12.48 µL of Adapter/Ligation Solution (Invitrogen®, *Eco*RI/*Mse*I adapters, 0.4 mM ATP, 10 mM Tris-HCl, 10 mM Mg-acetate, 50 mM K-acetate) and 0.52 µL of T4 DNA ligase (1 U/µL) before incubation for 2 h at 20 °C. Ligation solution was diluted to 1:6 and 4 µL were used to perform the pre-amplification by adding 16 µL of pre-amp primer mix (Invitrogen®), 2 µL of 10X *pfu* buffer with MgSO_4_ (Fermentas®) and 1 U of *pfu* DNA polymerase (Fermentas®) in a final volume of 24 µL.

Polymerase chain reaction was performed using a thermocycler (iCycler, Bio-Rad Laboratories). A first cycle was performed at 94 °C for 3 min, then 20 cycles were performed at 94 °C for 30 s, 56 °C for 60 s and 72 °C for 60 s, with a final cycle of 5 min at 72 °C. Three primer combinations with distinct polymorphic loci were selected for the selective amplification: E-CTA/M-ACA, E-CTA/M-AAC and E-CAA/M-ACA (Invitrogen). To assess reproducibility of the primer combinations, three different and independent individuals were repeated three times from the same DNA extraction for each combination. The mean reproducibility values for the three combinations were reasonably high (86.1–93.0 %). Amplifications were performed using 5 µL of 1:6 diluted pre-amplification reaction, adding 0.4 µL of dNTPs (10 mM), 2 µL of *pfu* buffer with MgSO_4_, 0.4 U of *pfu* DNA polymerase, 1 µL of *Eco*RI primers (100 mM) and 1 µL *Mse*I primer (50 mM) to a total volume of 20 µL. Amplifications were programmed for 1 cycle at 94 °C for 2 min, 10 cycles consisting of 20 s at 94 °C and 30 s at 66 °C and 2 min at 72 °C. The 66 °C annealing temperature of the 10 cycles was subsequently reduced by 1 °C every cycle, and continued at 56 °C for the remaining 20 cycles, with a final hold at 60 °C for 30 min.

Capillary electrophoresis of all samples was performed with the selective amplification products of AFLP on an automated 48-capillary DNA sequencer (MegaBACE^TM^ 500, GE Healthcare). Samples were prepared for analysis by diluting the final amplified product to 1:10. All samples included 1 µL of MegaBACE ET550-R DNA size standard (GE Healthcare) diluted at 1:6. Samples were run for 75 min using GT Dye Set 2 [ET-Rox, FAM, NED, HEX].

### Data analysis

The fragments amplified by AFLP primers were visualized using MegaBACE Fragment profiler v1.2 (GE Healthcare) and manually scored as either present (1) or absent (0). Fragments with lengths between 60 and 500 base pairs were included in the analysis. Estimates of allelic frequencies were computed using the square root method of the null homozygote frequency assuming Hardy–Weinberg equilibrium, as implemented in the program AFLP-SURV V1.0 (Vekemans *et al.* 2002). Genetic diversity within populations was estimated as the proportion of polymorphic loci at the 5 % level, and as Nei’s expected gene diversity (*H*_e_) that averages expected heterozygosity of the marker loci (Lynch and Milligan 1994). To test for effects of genetic drift on reproduction, we correlated mean mass of seeds (available for 17 of the populations; see Maurice *et al*. 2012) with the proportion of polymorphic loci and *H*_e_ in the populations.

The genetic structure within and among populations was analysed on the basis of AFLP allele frequencies using the square-root method implemented in AFLP-SURV V1.0 assuming that the populations were in Hardy–Weinberg equilibrium, to calculate an overall *F*_ST_ value following the treatment by Lynch and Milligan (1994) with 1000 permutations. We also performed a separate analysis with AFLP-SURV for each altitude class.

The genetic structure of *A. montana* was studied at the landscape level using a Bayesian clustering method to infer population structure and assign individuals to geographical regions, as implemented in STRUCTURE V2.3 (Pritchard *et al.* 2000) which allows the analysis of dominant data (Falush *et al.* 2007). We used a model of no population admixture for the ancestry of the individuals without prior information about the regional membership of the populations and assumed that the allele frequencies are correlated within populations. We conducted a series of 11 independent runs for each value of *K* (the number of clusters) between 1 and 30 in order to quantify the amount of variation of the likelihood of each *K*. We found that a length of the burn-in and Markov chain Monte Carlo (MCMC) of 10 000 each was sufficient. Longer burn-in or MCMC did not significantly change the results. The model choice criterion implemented in STRUCTURE to detect the *K* most appropriate to describe the data is an estimate of the posterior probability of the data for a given *K*, Pr(*X*|*K*) (Pritchard *et al.* 2000). This value is called ‘Ln P(*D*)’ in STRUCTURE, which we refer to as *L*(*K*) afterwards. An ad hoc quantity based on the second-order rate of change of the likelihood function with respect to *K* (Δ*K*) did show a clear peak at the true value of *K* (Evanno *et al.* 2005). We calculated Δ*K * = * m*(|L(*K* + 1) −2L(*K*) + L(*K*−1)|)/SD[L(*K*)] where *m* is the mean and SD the standard deviation. The best estimate of *K* was defined by the model giving the highest probability of the data, with a peak in the Δ*K* graph, and which also gave consistent results over multiple runs. Finally, the runs of the STRUCTURE simulation were aligned using the FullSearch option with the cluster matching and permutation program CLUMPP V1.1.2 (Jakobsson and Rosenberg 2007).

A hierarchical analysis of molecular variance (AMOVA) was used to partition the genetic variability among colline and montane Vosges populations, populations within population groups and individuals as implemented in GenAlEX 6.41 (Peakall and Smouse 2006). The variance components from the analysis were used to estimate Φ-statistics which are similar to *F*-statistics (Excoffier *et al.* 1992).

We identified non-neutral markers with the program BAYESCAN (Foll *et al*. 2008), removed them from the dataset, and ran a second AMOVA. The false discovery rate (FDR) in BAYESCAN was set to 0.001 (see Foll *et al*. 2008). The method used by BAYESCAN 2.01 was found to be robust against deviations from the island model and yielded very few false positives in all simulations in a recent study comparing several methods for detecting markers under selection (De Mita *et al*. 2013).

We obtained the following bioclimatic variables for each study site (representative of 1950–2000) in a grid-size of about one square kilometre (30 arc seconds) from the Worldclim database version 1.4. (Hijmans *et al.* 2005; www.worldclim.org): annual mean temperature, temperature seasonality, temperature annual range, temperature of driest quarter, and annual precipitation. Because these variables were intercorrelated, we identified two principal components (PCs) by PCA with varimax rotation (SPSS 19.0). PC ALTI explained 45.6 % of the variation and was highly correlated with annual precipitation (*r*  =  0.97) and mean annual temperature (*r*  =  −0.96), indicating that PC ALTI corresponded to a climatic gradient related to altitude. PC CONTI explained a further 33.5 % of the variation and was highly correlated with temperature seasonality (*r*  =  0.98) and temperature annual range (*r*  =  0.96); indicating that PC CONTI corresponded to a gradient in continentality.

We then studied the relationship between the frequency of the identified non-neutral markers and PC ALTI and PC CONTI with a generalized linear model with a logit link and a quasibinomial error distribution (see Crawley 2009), using the glm package of R version 3.0.1. To correct for spatial autocorrelation, we included latitude and longitude in the model. Moreover, the first two components of a PCA ordination of the neutral AFLP loci were also added to the model to correct for the genetic structure present in the neutral model. McFadden’s Pseudo *R*^2^ was estimated as the ratio among the log-likelihood of the model of interest and the log-likelihood of the null model.

Pairwise Φst genetic distances among (1) all pairs of populations, (2) separately for the colline and the montane Vosges populations and (3) for a subset of the colline populations whose geographical distances were similar to those of the montane Vosges populations were related to geographical distances and the significance of the relationships tested with a Mantel–Test implemented in GenAlEX (1000 permutations).

## Results

### Climatic characteristics of the study sites

**Figure 2.**
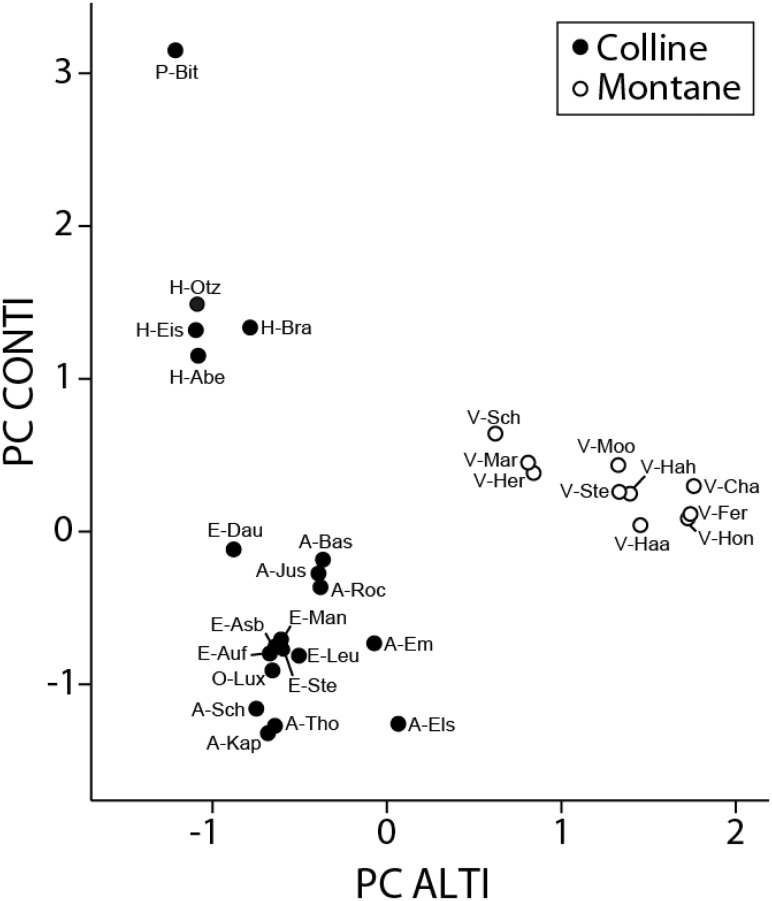
Principal component analysis of five bioclimatic variables extracted for 30 populations of *A. montana* from the Worldclim database version 1.4. (Hijmans *et al.* 2005; www.worldclim.org): annual mean temperature, temperature seasonality, temperature annual range, temperature of driest quarter, and annual precipitation. The first factor PC ALTI was highly correlated with annual precipitation (*r* = 0.97) and mean annual temperature (*r* = −0.96), indicating that PC ALTI corresponded to a climatic gradient related to altitude. PC CONTI was highly correlated with temperature seasonality (*r* = 0.976) and temperature annual range (*r* = 0.96); indicating that PC CONTI corresponded to a gradient in continentality. For abbreviations of population names see Table 1.

The clusters identified by the PCA-analysis of the bioclimatic variables corresponded well to the three geographical regions (Table 1 and Fig. 2). A first cluster consisted of the four populations of the Hunsrück region and the one Pays de Bitche population characterized by a warm climate with relatively low precipitation, high annual temperature range and high temperature seasonality. The second cluster corresponded to the populations of the Ardennes–Eifel region characterized by lower temperature and precipitation. Our results thus indicate that the Hunsrück populations grow in a climate different from that of the Ardennes–Eifel populations, although they are at the same altitudinal level (Table 1). The third cluster corresponded to the ten populations of the Vosges mountains characterized by a cold and wet climate with relatively high temperature seasonality.

### Genetic diversity within populations

Using three primer combinations, 399 AFLP bands were scored with no private bands specific to a population. All 494 individuals had a unique multilocus genotype. The proportion of polymorphic loci (PPL) in the 30 populations ranged from 46.6 to 86.7 % (Table 1). The proportion of polymorphic loci varied strongly among regions (*F*_2,27 _ = _ _14.55, *P* < 0.001) and was higher in the Ardennes–Eifel region (75.1 %) than in the Hunsrück (59.2 %) and Vosges region (62.4 %). The proportion of polymorphic loci in the montane Vosges populations was lower than in the colline populations (62.4 % vs. 71.2 %; *F*_1,28 _ = _ _6.19, *P* < 0.05).

**Figure 3.**
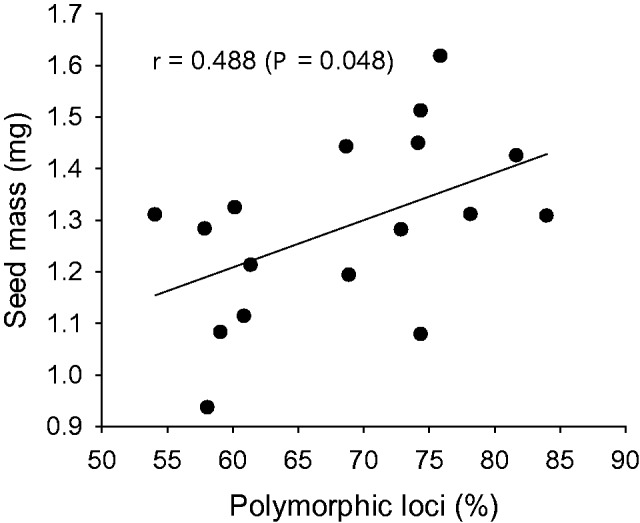
The relationship between mean seed mass in 17 populations of *A. montana* and the proportion of polymorphic loci.

The mean value for Nei’s genetic diversity within populations (H_e_) assuming Hardy–Weinberg equilibrium was 0.210. Genetic diversity was 10.2 % lower in montane Vosges (H_e _ =  0.195) than in colline populations (*H*_e _ =  0.217, *F*_1,28 _ = _ _5.86, *P* < 0.05), and differed significantly among the three geographical regions Ardennes–Eifel, Hunsrück and the Vosges mountains (*F*_2,27 _ = _ _35.16, *P* < 0.001). Among the colline regions, mean genetic diversity of the populations in the Hunsrück region was significantly lower than that of the populations of the Ardennes–Eifel region (H_e_  =  0.178 vs. H_e_  =  0.231, *F*_1,18 _ = _ _42.3, *P* < 0.001). In multiple regressions relating the measures of genetic diversity to altitude and (log)population size, separately for the three regions, Nei’s gene diversity was not significantly related to the two explanatory variables in any of the regions. However, adjusted for the effects of altitude, the number of polymorphic loci significantly increased with population size in the Ardennes-Eifel region (*β*  =  0.83, *t*  =  3.65, *P* < 0.01). Seed mass increased with the proportion of polymorphic loci (Fig. 3), but not with H_e_ (*r*  =  0.287, *P*  =  0.265).

### Population genetic structure

**Figure 4.**
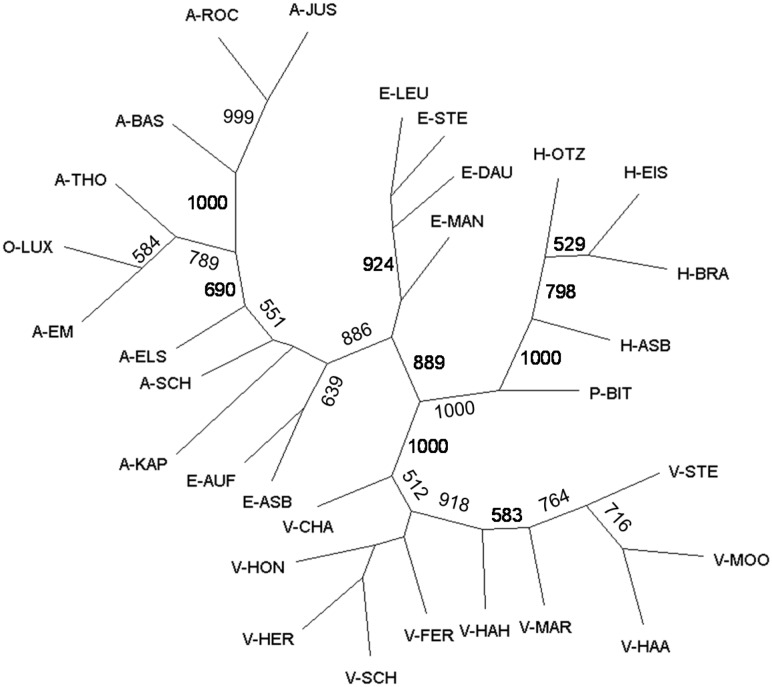
Neighbour-joining tree of 30 populations of *A. montana* based on Nei’s genetic distances derived from AFLP markers. Numbers near the branches indicate bootstrap values above 500 of 1000 bootstraps. For abbreviations of population names see Table 1.

Using the modal value of Δ*K* rather than the maximum value of L(*K*) allowed us to identify with STRUCTURE several groups corresponding to the uppermost hierarchical level of partitioning between populations. The highest modal value of Δ*K* was at *K*  =  3, corresponding to the number of geographical regions. There was a nearly complete correspondence between the clusters identified by STRUCTURE and the three geographical regions (Table 1). A first cluster consisted of the populations of the Ardennes–Eifel region and the Pays de Bitche population, which showed some admixture between the Ardennes–Eifel and the Hunsrück regions. The second cluster corresponded to the four populations of the Hunsrück region. The third cluster corresponded to the ten populations of the Vosges mountains. The proportion of membership of the individuals of the populations in each of the three identified clusters ranged from 0.901 to 1.000 (Table 1). The neighbour-joining tree based on Nei’s genetic distance revealed a clustering pattern similar to the clusters identified by STRUCTURE (Table 1). However, the population with lowest elevation in the Pays de Bitche region (P-Bit; 281 m a.s.l.), was more related to the Hunsrück region in the neighbour-joining tree (Fig. 4). Furthermore, the neighbour-joining tree indicated that populations from the south-west of Belgium (A-Jus, A-Bas and A-Roc), and from the north of Luxemburg and neighbouring E-Belgium (A-Tho, A-Lux and A-Em), formed two separate sub-groups within the Ardennes and Oesling region in concordance with the geographical position of the sampled populations (Figs 1 and 4).

The estimate of overall *F*_ST_ obtained by AFLP-Surv assuming Hardy–Weinberg equilibrium was lower (0.122 ± 0.11) than the value obtained by the AMOVA (Ф_ST_  =  0.159). Results of the AMOVA showed that there was a significant genetic differentiation between the Vosges and the colline region (8.2 %) and among the populations within the groups (7.8 % of total variation), although the largest part of the total genetic variation was due to differences between plants within populations (84 %, Table 2).

**Table 2.** Summary of analysis of molecular variance based on AFLP-analysis of 494 *A. montana* individuals from 30 populations. The genetic variation was partitioned between colline and populations in the Vosges mountains.

Source	df	Variance component	Variance (%)	*P*
Between Vosges and colline populations	1	5.32	8.2	< 0.001
Among populations within population groups	28	5.03	7.8	< 0.001
Within populations	464	54.45	84.0	< 0.001

**Figure 5.**
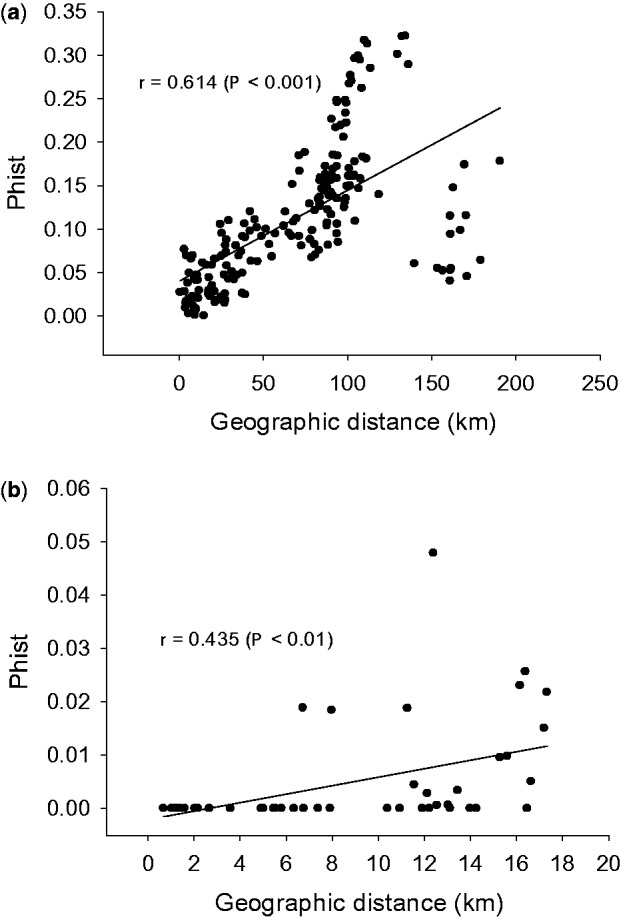
The relationship between the genetic and geographical distance between (a) 20 pairs of colline populations and (b) 10 pairs of montane populations of *A. montana*.

A separate analysis with AFLP-SURV for the colline and the montane Vosges populations indicated that the genetic differentiation among colline populations (*F*_ST_  =  0.12) was much higher than that among Vosges populations (*F*_ST_  =  0.004). Overall genetic differentiation between all 30 populations (pairwise Ф_ST_) was related to their geographic distance (Mantel test, *r*  =  0.44, *P* < 0.01). This isolation by distance pattern was much stronger for the colline populations (Mantel test, *r * =  0.61, *P* < 0.001; Fig. 5a) than for the Vosges populations (Mantel test, *r * =  0.44, *P* < 0.01; Fig. 5b). However, in a subset of pairs of colline populations whose geographical distances were similar to those separating the Vosges populations (< 27 km), the IBD pattern among colline populations was much weaker (*r * =  0.21 *P*  =  0.18).

### Putative selective loci

Using the program BAYESCAN 2.01, 63 loci (15.8 %) were identified as outliers with FDR values below 0.001. Divergence of 49 loci (77.8 %) was higher and that of 14 (22.2 %) significantly lower than under a neutral expectation indicating that directional selection occurred at a higher frequency than stabilizing selection.

Multiple logistic regressions revealed that of the 63 putative selective loci, 44 showed a significant (*P* <0.05) relationship with one or two of the principal components derived from a PCA of bioclimatic variables. After correcting for spatial autocorrelation and for the genetic structure of neutral loci two loci were significantly related to climatic PCs in analyses of deviance, suggesting that these loci may be adaptive and their frequency related to climatic conditions (Table 3). However, other environmental factors like soil conditions that were not studied, but vary among populations could also be responsible for the differences in the frequency of putatively adaptive loci.

**Table 3.** Intercepts and regression coefficients from multiple logistic regression analyses of the relationship between the frequency of two putative loci under selection in populations of *A. montana* and two principal components describing bioclimatic variables. PC ALTI corresponded to a climatic gradient related to altitude (annual precipitation and mean annual temperature) and PC CONTI to a gradient in continentality (temperature seasonality and temperature annual range), to correct for spatial autocorrelation latitude and longitude were included in the model. To correct for the genetic structure present in the neutral model the first two components of a PCA ordination (DIM1 and DIM2) of the neutral AFLP loci were also added to the model. *, *P* < 0.05; **, *P* < 0.01. Bp, fragment size expressed as number of base-pairs. McFadden’s pseudo *R*^2^ for the models is also indicated (see text for details).

Dependent variable (locus)	bp	*R*^2^	Intercept	Explanatory variable	Estimate	*t*-value	
E-CTA/M-ACA							
B1-37	96	0.891	52.167	PC ALTI	−0.237	−0.534	
				PC CONTI	1.132	2.958	**
				lat	−1.005	−1.472	
				long	−0.206	−0.327	
				DIM1	0.001	0.012	
				DIM2	−0.115	−1.673	
E-CTA/M-AAC							
B2-30	89	0.933	105.417	PC ALTI	1.231	2.466	*
				PC CONTI	1.941	3.371	**
				lat	−1.983	−1.533	
				long	−1.247	−1.516	
				DIM1	−0.001	−0.020	
				DIM2	0.024	0.266	

A second AMOVA, using a reduced data set with the 63 non-neutral molecular markers removed, resulted in lower Φ_ST_ values than the analysis using the complete dataset (Φ_ST_  =  0.12 vs. Φ_ST_  =  0.16). This was also the case when the variation was partitioned among the colline and the montane Vosges populations (Φ_ST_  =  0.06 vs. Φ_ST_  =  0.08), suggesting that not only genetic drift but also divergent selection has influenced the genetic differentiation among the colline and montane Vosges populations.

## Discussion

### Genetic diversity within populations

We found that large *A. montana* populations still exist at both altitudinal levels. Although colline populations are much more isolated than the montane Vosges populations, even most small colline populations have conserved a considerable amount of genetic diversity. Due to their isolation, current gene flow among most colline populations is probably very low, but the effects of genetic drift in small populations are not yet very pronounced, as there was no clear relationship between genetic diversity and population size. However, we only could estimate the number of rosettes in the populations, and the relationship between the number of genets and rosettes in this clonal plant is not known and might vary strongly among populations. A prevailing assumption has long been that sexual recruitment is rare in clonal plants implying low genetic diversity, but an increasing number of studies indicate that populations of clonal plants may maintain considerable amounts of genetic diversity (Holderegger *et al.* 1998; Bengtsson 2003; Pluess and Stöcklin 2004).

Our finding of considerable genetic variation in the populations is in agreement with the situation of *A. montana* in the Rhön mountains (Kahmen and Poschlod 2000). Fragmented populations of long-lived plant species like *A. montana* may conserve high levels of genetic diversity for a long time, especially if the surviving plants are remnants of formerly large, well-connected populations (Honnay and Bossuyt 2005; Beatty *et al.* 2008). In contrast, in the Dutch populations of *A. montana* studied by Luijten *et al.* (2000) genetic variation was very low. In the Netherlands, fragmentation of *A. montana* populations is far more pronounced and many of the populations studied by Luijten *et al.* (2000) were very small. However, comparing the results from our AFLP study to those of Luijten *et al.* (2000) and Kahmen and Poschlod (2000) is difficult because the types of markers differ (Garcia *et al.* 2004; Nybom 2004).

We found a positive relationship between mean seed mass and the proportion of polymorphic loci in 17 populations of *A. montana*, but no positive relationship with population size estimated by the number of rosettes. This suggests inbreeding effects in low diversity populations. Similarly, in the Netherlands, several components of fitness were significantly related to population size in *A. montana* (Luijten *et al.* 2000).

### Genetic differentiation among populations

The analysis of the genetic population structure revealed significant genetic differentiation between the Vosges and the colline populations and among populations within regions. Overall, the genetic differentiation among the studied *A. montana* populations was moderate (AMOVA, *F*_ST_  =  0.16) in comparison to that of other species studied using dominant markers (Nybom 2004). Genetic differentiation among the colline populations was higher than among the montane Vosges populations, but this was due to the greater distances among the studied colline populations, as genetic distance increased with geographical distance. This is in accordance with an isolation by distance model (IBD) where geographically closer populations are connected more efficiently by gene flow (Lowe *et al.* 2004). While at a similar small scale, there was a clear IBD pattern for the montane Vosges but not for the colline populations, at a large scale, there was a strong IBD-pattern for the colline populations. The significant IBD pattern at the large scale is likely to be a legacy of historical gene flow, whereas the lack of IBD at shorter ranges is most likely caused by random genetic drift after fragmentation reduced gene flow more recently. At the beginning of the 20^th^ century, large areas of the region were still covered by heathland and nutrient-poor grassland communities (Hoyois 1949; Dumont 1979), which were suitable habitats for *A. montana* (Maurice *et al.* 2012). Moreover, populations of long-lived plants like *A. montana* may be buffered against the effects of fragmentation due to their long generation times (Honnay and Bossuyt 2005; Beatty *et al.* 2008, Walisch *et al.* 2015).

The STRUCTURE analysis indicated three groups of populations that were separated genetically from each other: those in the Vosges mountains, in the Hunsrück and in the Ardennes-Eifel region. The three groups of populations have probably been isolated for a long time, because the old Rhenish massif of the Hunsrück is separated from the Ardennes–Eifel region by the deep Moselle river valley, and the two colline groups from the Vosges mountains by the Saar river valley. As *A. montana* is a characteristic plant of open heathlands and nutrient-poor acidic grasslands (Maurice *et al.* 2012), the nutrient-rich riparian habitats of the Mosel and Saar river valleys could have isolated the groups of populations. Overall, the results suggest that the current population structure of *A. montana* can be described as regional ensembles of populations with more recent historical gene flow within regions. However, it is likely that at least some part of the genetic differences among regions is due to differences in allele frequencies of non-neutral markers.

### Non-neutral markers

The genome scan study of the 30 populations of *Arnica montana* showed that after controlling for spatial autocorrelation and patterns of neutral variation, two AFLP-loci strongly correlated with the two bioclimatic principal components representing a climatic gradient with altitude (annual precipitation and mean annual temperature) and a gradient in continentality (temperature seasonality, temperature annual range), which suggests that these molecular markers may be under directional selection. These results could indicate that populations of *A. montana* are adapted to the local climatic conditions. Although this is only correlative evidence and the observed pattern could also be due other environmental factors, local adaptation in response to local climatic conditions has frequently been found in plants (Becker *et al.*, 2006; Leinonen *et al.*, 2009).

## Conclusions

Our results indicate that in contrast to our expectation even strongly fragmented colline populations of *A. montana* have conserved a considerable amount of genetic diversity. In the short term, habitat destruction and deterioration, and not genetic erosion, are the strongest threats to both colline and montane populations. For colline populations, eutrophication through aerial deposition of nitrogen and influx from neighbouring fertilised fields negatively affects habitat suitability for *A. montana* (Maurice *et al.* 2012).

However, without suitable management measures, populations will continue to decrease in size (Maurice *et al.* 2012) and lose genetic diversity due to random genetic drift as already seen in the Netherlands (Luijten *et al.* 2000) and affect population persistence in the long term. In order to preserve actual genetic diversity, suitable management measures aimed at reducing eutrophication and increasing the size of small populations are necessary. Management measures such as turf cutting could enhance seedling recruitment in small colline *A. montana* populations (Knapp 1953, Vergeer *et al.* 2005) and thus preserve genetic variability.

The strong genetic differentiation found between colline and montane Vosges populations probably precludes the use of plants from montane populations for the reintroduction of *A. montana* or the reinforcement of populations in the lowlands. Moreover, results of a genome scan study indicated differences in loci under selection, suggesting that plants from montane Vosges populations could be maladapted to conditions at colline sites. There could also be a considerable risk of outbreeding depression. Our results suggest caution in using material from montane populations of rare plants for reinforcement of small genetically depauperate lowland populations.

## Sources of Funding

The project was supported by the National Research Fund of Luxembourg (Ref. BFR07 TR-PHD-054) and by the Musée National d’Histoire Naturelle, Luxembourg.

## Contributions by the Authors

T.M., G.C., D.M. and S.M. designed the study. T.M. carried out the practical work. T.M., G.C. and D.M. analysed the data, T.M., G.C., D.M. and S.M. wrote the manuscript.

## Conflicts of Interest Statement

None declared.
